# Reports of Cases

**Published:** 1872-05

**Authors:** Carlisle Terry

**Affiliations:** Columbus, Georgia


### REPORTS OF CASES.

BY CARLISLE TERRY, M.D., COLUMBUS, GEORGIA.

[Presented to the Georgia Medical Association, at its Annual Meeting, in Columbus, 1872.]

### *THREE CASES OF GUN-SHOT WOUNDS OF THE BRAIN.*

The following cases, never having been reported, may prove of interest, as showing how severely the brain may be injured, and recovery follow.

CASE I.—Occurred at the battle of Shiloh, on the 7th April, 1862. A boy about eighteen years old was brought to me at Shiloh Church, who had been shot while lying down on the skirmish line. A minnie ball had entered his head a little to the left of the sagittal suture, midway between the coronal and lambdoidal, and passed downward and backward to the base of the brain. I passed my finger into the orifice its full length, but could not reach the ball. However, I removed some spiculæ, and calling the attention of Dr. J. C. Nott, who was operating near me, to it, we both concluded that it was a fatal case, and merely ordering cold-water applications, left it.

To my surprise, some three days after, the boy was brought more than twenty miles over a terrible road, to Corinth, and put in my hospital. I placed him in Ward A, under charge of Assistant Surgeon Frank Hawthorne. His right side was then paralyzed, and speech somewhat impeded. The brain wound discharged largely of pus and brain matter; but under the careful treatment of Dr. Hawthorne, he gradually improved; the wound closed; and when we evacuated in May (27th), he was walking about, with only a stick, and was sent home with a discharge.

CASE II.—On Sunday, April 7th, 1862, J. R———, at the battle of Shiloh, was struck just over the left eye with a rough iron canister-shot, which penetrated the brain, but stopped just inside the skull. On the fifth day after, he was brought *in a road-wagon*, over this same bad road, to the hospital. His pulse was strong and full, at forty to the minute; pupils largely dilated, but mind perfectly clear; appetite good; courage firm, and no paralysis.

The next day, in the presence of Drs. Foard, Stone, Choppin, and a number of others, I removed the shot. He stated that several attempts had been made on the field; but as upon the slightest touch, brain matter would pour out, and the ball give way, it had been left as it was. Turning back flaps of the frontal muscles, I pinches off pieces of the brow—as the orifice appeared to be smaller than the shot—until I could introduce a scoop and throw out the ball, which presented a very rough ridge around it where the wounds had imperfectly closed, and a neck on each side, where it had been broken from others. With the shot, came out some two or three table-spoonsful of brain matter and many spiculæ. The wound was simply dressed, and the cure progressed favorably. The young man was walking about his ward in less than a week, although his pulse did not rise to its natural standard for some time longer. In three weeks, he was sent home to Mississippi, apparently well, except that the wound still discharged some.

Case III.—Occurred at the siege of Corinth. J. S. R——, private in Wirt Adams' cavalry, was shot on the 18th of May, 1862, while on duty before Corinth, some six miles from town. was brought in an ambulance after night, and deposited at the Tishamingo Hotel Hospital; but having before been in the Academy Hospital, and as the driver would not bring him on, he walked one-quarter of a mile over a bad corduroy road, in the dark, and arrived, at eleven P.M., at the hospital.

Examination disclosed that a minnie ball had entered the frontal bone at very nearly the center of the forehead. The index finger could readily be passed its whole length toward the occipital bone, so that the ball must have lodged against that bone. The wound was cleared of spiculæ and loose brain matter, and cold-water dressing applied. As in Case II., the pulse was slow and full (forty-five, I believe), but no paralysis or mental disturbance. Good appetite, and spirits buoyant. Case progressed very favorably until we evacuated, on the 27th, when he was sent to Jackson, Mississippi, without a bad symptom, and the case passing out of my observation, I never heard the termination. His chances, however, appeared much better than those of Case I., and I have no doubt but that his recovery was perfect.

---

### *CASE OF SUPERFŒTATION.*

On the morning of the 5th of July, 1870, I was called to a lady of this city, Mrs. M———. Upon arrival, at nine A.M., found her in labor, with frequent and regular pains. I asked her if it was her full time, according to her calculations. "Yes," said she, "I was unwell on the first of October, and ceased to menstruate on the third. I missed November and December; but on the 15th of December, I was unwell a little, and thought I was about to miscarry, but after a little *show*, it passed off."

Upon further inquiry, I found that she had been taken with pains soon after aring at six o'clock, and that with the second pain, the waters broke, discharging, as she thought, a full supply. Vaginal examination disclosed the head occupying the lower passage, and after a few pains, a boy was born. Very evidently, *not* a full seven-months' child; no hair; very imperfect nails; skin very thin and tender; skull very soft; fontanels large. While tying the cord, the placenta was expelled.

Upon examining the abdomen of the mother, I found it very little, if any, diminished in size; and on vaginal examination, found a large and strong bag of water presenting, and filling the pelvis. Pains continued with increased severity, and in ten or fifteen minutes the membranes ruptured, and another and *larger* head presented. Directly, a large and well-formed girl was born. The child was fully matured, with full head of hair, firm skull, and well-formed nails. Another and much larger placenta followed soon.

The first-born child weighed scant four pounds; the second, full eight pounds. The former lived six days, sleeping nearly all the time, and presenting all the characteristics of a prematurely-born babe; the latter, at the time of writing—September, 1870—is well, and a promising child.

Mrs. M—— had borne three children previously, and had had natural and easy labors. I found, also, that her husband—whose business required his frequent absence—had returned and spent several nights with her at the time in December when she had the "show," and immediately subsequent.

The facts in this case are so plain, and appear to prove that a second impregnation occurred, nine or ten weeks after the first, so conclusively, that I can not see how any other theory can stand at all.

